# Antibiotic prophylaxis and infectious complications in patients on peritoneal dialysis undergoing lower gastrointestinal endoscopy

**DOI:** 10.1093/gastro/goaa017

**Published:** 2020-04-25

**Authors:** William T Clarke, Venkata R Satyam, David I Fudman, Samantha Zullow, Kashika G Goyal, Katerina L Byanova, Christopher Huang, Joseph D Feuerstein

**Affiliations:** Division of Gastroenterology, Department of Medicine, Beth Israel Deaconess Medical Center, Harvard Medical School, Boston, MA, USA; Department of Medicine, Boston Medical Center, Boston University School of Medicine, Boston, MA, USA; Division of Gastroenterology, Department of Medicine, Beth Israel Deaconess Medical Center, Harvard Medical School, Boston, MA, USA; Present affiliation: Division of Digestive and Liver Diseases, Department of Internal Medicine, University of Texas Southwestern Medical Center, Dallas, TX, USA; Department of Medicine, Boston Medical Center, Boston University School of Medicine, Boston, MA, USA; Present affiliation: Division of Gastroenterology and Hepatology, New York University Langone Medical Center, New York University School of Medicine, New York, NY, USA; Department of Medicine, Beth Israel Deaconess Medical Center, Harvard Medical School, Boston, MA, USA; Department of Medicine, Beth Israel Deaconess Medical Center, Harvard Medical School, Boston, MA, USA; Section of Gastroenterology, Department of Medicine, Boston Medical Center, Boston University School of Medicine, Boston, MA, USA; Division of Gastroenterology, Department of Medicine, Beth Israel Deaconess Medical Center, Harvard Medical School, Boston, MA, USA

## Introduction

Peritoneal dialysis (PD) is widely used for the treatment of end-stage renal disease. Patients treated with PD have similar outcomes to those treated with hemodialysis [1]. However, PD-related infections including peritonitis have been reported at 1.66 episodes per patient per year [2]. One possible source of peritonitis is gastrointestinal (GI) endoscopic procedures. In 2005, the International Society for Peritoneal Dialysis (ISPD) published guidelines suggesting antibiotics be given to PD patients prior to colonoscopy [3]. This suggestion was mostly based on expert opinion and a theoretical concern that patients undergoing colonoscopy are at increased risk for enteric peritonitis from bacterial translocation across the bowel wall. While there is no direct evidence to support this theory, case reports have suggested a risk of transient bacteremia associated with colonoscopy.

A retrospective study from Hong Kong published in 2007 identified 77 PD patients who had undergone 97 colonoscopies [4]. In 18 cases, antibiotic prophylaxis was given and no episodes of peritonitis were identified. In 79 cases in which antibiotics were not given, there were five episodes (6.3%) of peritonitis. Further episodes of colonoscopy-related peritonitis have been reported [5, 6]. The 2016 update to the ISPD guidelines suggests the use of antibiotic prophylaxis prior to colonoscopy [7]. In 2015, the American Society for Gastrointestinal Endoscopy (ASGE) guidelines made a suggestion based on low-quality evidence that PD patients undergoing lower GI endoscopy receive antibiotic prophylaxis [8]. Given the limited data available to support these guidelines, we sought to determine the frequency with which periprocedural antibiotic prophylaxis are given and to identify the rate of infection in PD patients undergoing lower GI endoscopy.

## Patients and methods

We retrospectively identified all patients over age 18 on PD who underwent sigmoidoscopy or colonoscopy between 1998 and 2017. This study was performed at Beth Israel Deaconess Medical Center and Boston Medical Center and approved by the institutional review boards at both institutions.

We recorded patient demographics, procedure characteristics, and periprocedural antibiotics. Charts were reviewed for any outpatient or inpatient reports of intra-abdominal infections within 14 days of the endoscopy. Any suspected cases of peritonitis are reported. Peritonitis was classified as confirmed if the peritoneal-fluid white-cell count was >100 cells/mm^3^, if there was any positive bacterial or fungal culture of the peritoneal fluid or if there was a positive gram stain [9].

## Results

We identified 127 colonoscopies and sigmoidoscopies performed on 99 PD patients at two large tertiary-care academic medical centers. The patients were predominantly male (63%) and the median age was 60 (interquartile range, 54–66) years. Most procedures were colonoscopies (93%) and performed in the outpatient setting (79%). Colon-cancer screening was the most frequent indication (63%). Interventions were performed in 90 procedures (71%) including 40 snare polypectomies, 33 forceps polypectomies, and 30 biopsies (Table 1).

**Table 1.** Characteristics of peritoneal dialysis patients undergoing lower gastrointestinal endoscopies

Characteristic	No. of cases (%)
Age	
<40	2 (2%)
40–49	6 (5%)
50–59	37 (29%)
60–69	49 (39%)
70–79	25 (20%)
≥80	8 (6%)
Male gender	80 (63%)
Ethnicity	
Asian	11 (9%)
African American	60 (47%)
Hispanic	22 (17%)
Caucasian	32 (25%)
Other	2 (2%)
Procedure type	
Colonoscopy	118 (93%)
Sigmoidoscopy	9 (7%)
Indication	
Screening, history of polyps, or family history	80 (63%)
Gastrointestinal bleeding	21 (17%)
Diarrhea	13 (10%)
Other	13 (10%)
Inpatient procedure	27 (21%)
Fellow involved in procedure	40 (31%)
Antibiotics given	14 (11%)
For prophylaxis only	10 (8%)
Already on antibiotics	4 (3%)
Endoscopic intervention performed	90 (71%)
Snare polypectomy	40
Forceps polypectomy	33
Biopsies	30
Other	9
Peritonitis	
Confirmed by peritoneal fluid	0 (0%)
Suspected	1 (1%)

Periprocedural antibiotics were given before 14 procedures (11%). In 10 procedures (8%), patients received antibiotics for the sole purpose of prophylaxis. Four patients (3%) were already on antibiotics for pre-existing or suspected infections. The most commonly prescribed antibiotics were levofloxacin and metronidazole, both of which were used five times.

There were no cases of peritonitis confirmed by peritoneal fluid within 14 days of endoscopy. One patient who did not receive prophylactic antibiotics was suspected of developing post-procedural peritonitis and treated with antibiotics. This patient was a 60-year-old woman who underwent colonoscopy with biopsies for diarrhea. However, her peritoneal-fluid analysis did not meet any of the criteria for peritonitis.

## Discussion

In this retrospective multicenter study, we found a poor compliance rate of 8% with ISPD and ASGE suggestions for antibiotic prophylaxis in PD patients. However, we did not find any cases of confirmed peritonitis and only one case of suspected peritonitis. Our findings demonstrate a low rate of peritonitis and may suggest that the need for antibiotics is not necessary.

Patients with end-stage renal disease on PD have outcomes similar to those on hemodialysis but have high rates of PD-related infections. Since 2005, guidelines from the ISPD have suggested antibiotic prophylaxis for PD patients undergoing lower GI endoscopy [3]. The ASGE adopted similar suggestions in 2015 [8]. However, there are few data including no randomized–controlled trials to support these suggestions. When considering prophylactic antibiotics, one must also consider the risks associated with antibiotics including antibiotic-associated infections such as *Clostridium difficile* and development of multidrug-resistant organisms.

Compliance with guidelines in general is variable. While our study did not specifically focus on this question, it is unclear why providers are not providing antibiotic prophylaxis. There are a number of possible explanations, such as other guidelines suggesting low rates of infection in GI procedures. For example, cardiology guidelines recommended against antibiotic prophylaxis for endocarditis in patients undergoing colonoscopy [10]. Alternatively, given the underlying weak supporting evidence, it is possible that clinicians disagree with the suggestions. Finally, many providers may not even be aware of the ASGE and ISPD guidelines.

Our study has a number of strengths and weaknesses. This is a multicenter study and is the largest cohort study to date on PD patients undergoing lower GI endoscopy. The multicenter design should reduce single institutional practice bias. However, our study does have limitations. It is a retrospective observational study, so it is possible that data such as admissions at other hospitals for peritonitis may have been missed. Additionally, there are relatively few PD patients undergoing lower GI endoscopy every year, so it took many years at two institutions to identify 99 patients undergoing 127 endoscopies. A much larger, multicenter, prospective trial is necessary to understand the risk of peritonitis in PD patients undergoing lower GI endoscopy and determine the benefits and risks of prophylactic antibiotics.

In summary, our study demonstrates a very low rate of antibiotic prophylaxis in PD patients undergoing lower GI endoscopy, but importantly also does not show any confirmed cases of peritonitis. Our data raise questions as to whether guidelines recommending antibiotic prophylaxis are warranted. However, further well-designed prospective studies are needed to determine whether antibiotics have any role in PD patients undergoing lower GI endoscopy.

## Authors’ contributions 

Study design and concept: JDF, WTC, CH. Acquisition of Data: WTC, SZ, KLB, KGG, DIF, VRS. Statistical analysis: WTC. Manuscript preparation and revisions: WTC, SZ, KLB, KGG, DIF, VRS, CH, JDF. Study oversight: JDF, CH.

## Funding

None. 
